# Identifying biologically relevant putative mechanisms in a given phenotype comparison

**DOI:** 10.1371/journal.pone.0176950

**Published:** 2017-05-09

**Authors:** Samer Hanoudi, Michele Donato, Sorin Draghici

**Affiliations:** 1 Department of Computer Science, Wayne State University, Detroit, MI, United States of America; 2 Department of Obstetrics and Gynecology, Detroit, MI, United States of America; Laboratoire Arago, FRANCE

## Abstract

A major challenge in life science research is understanding the mechanism involved in a given phenotype. The ability to identify the correct mechanisms is needed in order to understand fundamental and very important phenomena such as mechanisms of disease, immune systems responses to various challenges, and mechanisms of drug action. The current data analysis methods focus on the identification of the differentially expressed (DE) genes using their fold change and/or p-values. Major shortcomings of this approach are that: i) it does not consider the interactions between genes; ii) its results are sensitive to the selection of the threshold(s) used, and iii) the set of genes produced by this approach is not always conducive to formulating mechanistic hypotheses. Here we present a method that can construct networks of genes that can be considered putative mechanisms. The putative mechanisms constructed by this approach are not limited to the set of DE genes, but also considers all known and relevant gene-gene interactions. We analyzed three real datasets for which both the causes of the phenotype, as well as the true mechanisms were known. We show that the method identified the correct mechanisms when applied on microarray datasets from mouse. We compared the results of our method with the results of the classical approach, showing that our method produces more meaningful biological insights.

## Introduction

Identifying the mechanism involved in a particular phenotype is an essential step in understanding the biological phenomena that lead to the phenotype. Mechanism identification became more and more feasible with the availability of high-throughput biological data, which makes it possible to measure the expression of thousands of genes at once, and with the expanding knowledge of interactions between biological entities such as genes and proteins.

In biology, the concept of *mechanism* has several meanings [1]. In this paper, we are interested in the *causal mechanism* which is defined as “a step-by-step explanation of the mode of operation of a causal process that gives rise to a phenomenon of interest.” [1]. Henceforth, we will use the term *mechanism* to refer to the *causal mechanism*. We also use the qualifier “putative” because the mechanisms we identify here are not mechanistically proven, but rather proposed mechanisms compatible with all gene expression changes measured throughout the system.

Many of the existing approaches to identify biological mechanisms focus on the selection of differentially expressed (DE) genes. One basic approach is to consider the expression fold change between two groups (i.e. disease vs. healthy). Typically an arbitrary threshold on such fold change (FC) is applied, and a gene will be considered DE when its absolute FC value is greater than the chosen threshold [2–4]. Other approaches use genes expression to calculate a p-value for each gene representing the probability of obtaining the observed expression change just by chance. This p-value is then used alone or in conjunction with the FC value to determine if a gene is DE or not. The results obtained by these approaches are very sensitive to the selection of the FCs and p-values thresholds [5]. An important question here is related to the number of biological replicates needed and which RNA-seq differential expression tool should be used in order to identify the DE genes. Schurch and others [6] evaluated 11 tools on an RNA-seq experiment data with 48 biological replicates [7–19]. The false discovery rate was ≲ 5% for 9 out of the 11 tools for all numbers of replicates. Regardless of the tools used, these approaches only provide a set of DE genes. For most purposes, identifying a set of DE genes is useful but is far from providing an understanding of the underlying phenomena [20].

Several methods were developed to provide an understanding of the underlying biological mechanisms by building regulatory networks from scratch from gene expression data, [21–25] or by integrating gene expression with other molecular data types such as transcription binding sites, and protein-protein interaction (PPI) data [26–28]. These methods have the advantage of being able to go beyond the existing known pathways and discover completely new mechanisms. On the other hand, these methods are burdened by the need to re-discover and re-create every time networks corresponding to well understood phenomena, already described by existing gene signaling networks such as those contained in KEGG [29, 30], Reactome [31], and Pathway Commons [32].

This was the motivation behind developing a plethora of pathways analysis methods [33–44]. These methods avoid the burden of having to re-discover well understood mechanisms by using the existing databases of known pathways as the starting point and attempting to identify the mechanisms underlying a given phenotype by identifying which of the known pathways are significantly impacted by the given phenotype [34]. These methods are very valuable but many times, they cannot provide a full understanding of the underlying phenomena due to the sheer size of some of the existing pathways. For instance, many KEGG pathways [29, 30] can have well over one hundred genes (e.g. “Pathways in cancer” has 397 genes/nodes and 1107 edges/interactions). If one or more of these large pathways are identified as significantly impacted in a given phenotype, the researchers are still at a loss as to which part of such a large pathway is the real key to the given phenomenon. To relate to the previous example, most experiments comparing cancer samples vs. normal tissue are likely to identify the human “pathways in cancer” (hsa05200) as being significant without really helping the understanding of the mechanism underlying the particular type of cancer studied in that experiment.

In this paper we describe a method able to identify which specific gene-gene interactions and signals from an existing pathway may constitute the putative mechanism associated with a phenotype of interest. The approach presented here takes into account both the classical factors, i.e. the expression changes and statistical significance of genes, as well as the knowledge captured by all existing known pathways. In section *Materials and Methods* we describe the method in detail, and in section *Results* we present the results of the application of the method on several datasets. The results of the proposed method show it is capable of identifying the correct mechanism in all three mouse datasets included in the paper. We compare the results of our method with the results of the classical approach that identifies mechanisms based only on the genes’ fold changes and p-values, showing that our method goes beyond what can be obtained with the existing methods. The gene list generated with the proposed method ranked the KEGG pathways with the KO gene similarly or higher than the gene list of the classical approach when combined with the methods including ORA [45, 46], SPIA [38], and LEGO [39]. We applied the three methods ORA, SPIA, and LEGO, to show that the comparison with the classical approach is not favoring a particular pathway analysis method. We also show that the proposed approach is less sensitive to the choice of parameters compared with the classical methods of using FCs and p-values.

## Materials and methods

Here we are proposing a method, henceforth referred to as *HighEdgeS*, that is able to identify putative mechanisms in phenotype comparison experiments by incorporating gene expression values and previously known interactions among genes. This is achieved in the following four steps: First, the method builds a global graph that consists of all known interactions among genes. Here, we used interaction knowledge from the KEGG pathway database, which was downloaded on Dec 2016, although other pathway databases can be used. The second step is to assign scores to all edges in the global graph by taking into account the measured expression change of the genes connected to each edge, as well as the statistical significance of such change. To calculate gene expression measurements, we used custom chip definition files (CDFs) for the preprocessing of the microarray data [47]. Given two genes A and B, connected by an edge in the global graph, let us consider *FC*_*A*_ and *FC*_*B*_ as the expression changes of A and B, respectively, and *p*_*A*_, *p*_*B*_ the probability of observing *FC*_*A*_, *FC*_*B*_ just by chance. For each dataset, the FC value (*FC*_*A*_) for gene A was calculated by comparing the expression values in KO samples vs. normal samples. The p-value for gene A (*p*_*A*_) was also calculated between the same groups (i.e. KO samples vs. normal samples) using a moderated t-test. More specifically, the p-value and FC for all the genes, were calculated with the functions eBayes and topTable from the limma package in R [48] (see download_Normalize_FC.R file in the S1 Folder) The edge between A and B will have a score calculated as follows:

$$\begin{array}{c} EdgeScore_{AB}=\left|FC_{A}\right|\cdot \left(1-p_{A}\right)+\left|FC_{B}\right|\cdot \left(1-p_{B}\right) \end{array}$$ (1)

**Fig 1:**
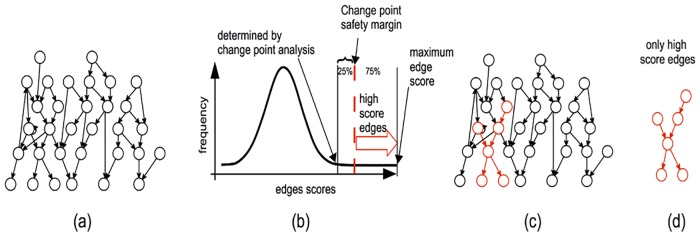
The workflow of the proposed method, HighEdgeS. a) The global graph constructed from all interactions present in all KEGG pathways; b) The edge score histogram constructed from the input data. A change point analysis [49] is used to determine the beginning of the flat area of the curve. The selected edges will be those in the top 75% of the remaining scores. c) The global graph showing the edges with high scores in red; d) The subgraph with the high scoring edges only representing the putative mechanism involved in the given phenotype.

After computing the score for each edge, the third step is to determine the “most important” edges. A basic strategy could be to choose a certain percentage of edges for instance, such as the top 1% or 5%. However, this strategy yields the same number of edges no matter what the input is, indicating them as relevant even in cases where there might not be any relevant edge (e.g. simulations of random input). Choosing an arbitrary threshold on the edge scores would have led to the same problem of sensitivity to parameters of the classical approaches. Therefore, we use a *change point analysis* [49] on the distribution of edge scores to *automatically* determine the critical edge score threshold based on the observed data. Intuitively, the change point can be thought of as the inflection point of the distribution of the edge scores after which the distribution becomes flat. An example is shown in Fig 1b.

Since we observed that the threshold determined by the change point analysis may still include a large number of edges, which may introduce some false positives, we decided to consider only the top 75% of the edges in this interval, thus leaving a *safety margin*. The 75% was chosen in order to avoid selecting a large number of genes, based on the typical distribution of the edge scores. No changes to this threshold were made after the initial choice. The interval of edge scores used to select edges is shown by the red arrow in Fig 1b. In the final step of this approach a graph representing the putative mechanism related to the phenotype is constructed from the high-score edges selected as above. The final result of the proposed analysis method is a graph with the following properties: a) many (but not all) nodes are genes with high fold changes and/or significant p-values, and b) each edge corresponds to a known biological signal (the initial graph is constructed using all known interactions from KEGG). The value here is identifying the small subset of interactions from the larger subsets of interactions described by curate pathways. Notably, the graphs constructed by this approach are generally different from the subgraphs that could be obtained based on fold-change alone, p-values alone or fold changes and p-values together.

The main problem when proposing a new analysis method is posed by the lack of gold standards for validation. In this case, our goal is to investigate whether the proposed analysis method is able to correctly identify the mechanism(s) involved in a given phenotype. In order to perform this type of validation we chose three datasets comparing a wild-type with a phenotype derived by knocking out (KO) a single gene. A knock-out dataset is a dataset where a gene is intentionally disrupted, inactivating it completely (or nearly so). In all these situations, we know the true cause of the phenomenon (the KO of the target gene), as well as the initial mechanism that causes the phenotype (the genes that are immediately downstream of the KO gene).

A good analysis method should identify the KO gene as central in the resulting mechanism, along with the interactions and processes downstream of the KO gene. However, since our approach uses an edge analysis, it may be seen more likely to yield networks of genes, which may be seen as an unfair advantage with respect to the classical approach that selects differentially expressed (DE) genes regardless of their connectivity. In order to level the field and ensure an unbiased comparison, we also compared the results of the two approaches using a classical pathway analysis that aims to identify the pathways that are implicated in a given phenotype.

We applied HighEdgeS, as well as the classical approach on three knock-out (KO) datasets. The KO genes in these datases were Myd88, NeuroD1, and Pxd1. The GEO [50] accession numbers for these datasets are GSE22873, GSE6030, and GSE29048, respectively. We compared the proposed approach with the classical approach by considering the entire list of genes provided by each method and using them to perform a pathway analysis, more specifically the over-representation analysis (ORA) [45, 46] SPIA [38], and LEGO [39]. In order to also compare the results of our method with a method that is not dependent on threshold selection, we used GAGE [40]. GAGE also ranks gene sets similarly to ORA, SPIA and LEGO, but its input is the entire set of genes in a dataset (i.e. not only the DE genes). In summary, the input to GAGE is: i) the entire set of genes in an experiment and ii) a list of gene sets (pathways with gene names only no interactions). GAGE’s output is *one* list of ranked gene sets/pathways. The results from GAGE were compared with the results obtained with the threshold automatically calculated by the proposed method. In both cases, we can calculate a false positive rate (FPR) and a true positive rate (TPR) for each data sets. For the methods SPIA, LEGO and GAGE, we used the R implementation versions 2.22.0, 1.0 and 2.20.1 respectively. In essence, we ask which of the known pathways are significantly enriched in the genes found by each of the two approaches (i.e. HighEdgeS and the classical). We chose to perform this comparison because, in principle, even a result in which the KO gene is not reported as relevant, but all the other genes related to the phenomenon are, should be considered a meaningful result. Pathways were considered significant when their FDR corrected p-values were < 0.1.

The assessment was performed considering that the pathways where the KO gene was present were truly causal for the phenotype and should be reported as significant (positives), whereas pathways without the KO gene should not be significant (negatives). Given this assumption, we can compute the number of True Positives, True Negatives, False Positives, and False Negatives obtained by analyzing each list of genes.

For HighEdgeS the list of relevant genes was the list of genes connected to at least one of the high-score edges, while for the classical approach it was the list of genes identified as DE by the choice of thresholds. In each case, we calculated the True Positive Rate (TPR) and False Positive Rate (FPR). Since the results of the classical approach are sensitive to the chosen thresholds, we calculated these measures for an entire range of thresholds. The positive likelihood ratio was computed across this entire range to determine the threshold that produced the best result within each dataset. For each dataset we considered the threshold that produces the best positive likelihood ratio in order to present the classical approach in the best possible light. In reality, using the fold change and p-value approach will always produce results inferior to the ones reported here because the optimal threshold cannot be known in advance for any given dataset.

Thresholds were generated as follows: for the classical approach the range starts from *log*_2_|*FC*| = 0.5 and −*log*_10_(*p*) = 0.5 (least stringent), to *max*(*log*_2_|*FC*|) and −*log*_10_(*p*) = 5 (most stringent) for each dataset. Note that the classical approach uses two variables: FC and p-value and requires an arbitrary threshold for each. In contrast, HighEdgeS does not require the user to choose any thresholds.

At each interval in the threshold range of HighEdgeS, the method was applied and the genes that were connected to the high score edges were used to rank pathways using ORA, SPIA and LEGO. The TPR and the FPR were calculated each time. A similar process was applied for the classical approach using the somewhat arbitrary, but commonly used, combinations of fold-change and p-value thresholds.

It is important to note that HighEdgeS returns a network, while the classical approach returns a set of genes. In order to make the results comparable of the two approaches: we built a network by retrieving all the interactions from KEGG connecting any pair of genes resulting from the classical approach. The comparison highlights the fact that in spite of the fact that both approaches use the same type of information (fold changes, p-values and known gene signals from KEGG), HighEdgeS is able to produce a sequence of interactions as the putative mechanism for the process involved in the phenotype, whereas the classical approach yields mostly disconnected genes. Note that the same values of *p*_*A*_, *p*_*B*_, *FC*_*A*_, and *FC*_*B*_ were used in both methods (i.e. the classical approach and HighEdgeS). Finally, because we used KEGG as a reference, HighEdgeS can only detect edges, and therefore genes, that are present in KEGG. In order to keep the comparison as fair as possible, for the classical approach we used only genes that belong to at least one KEGG pathway, as it would be unfair to compare connectivity if we were to use also genes that are disconnected because they do not belong to any KEGG pathway.

## Results

### Myd88 knock-out

**Fig 2:**
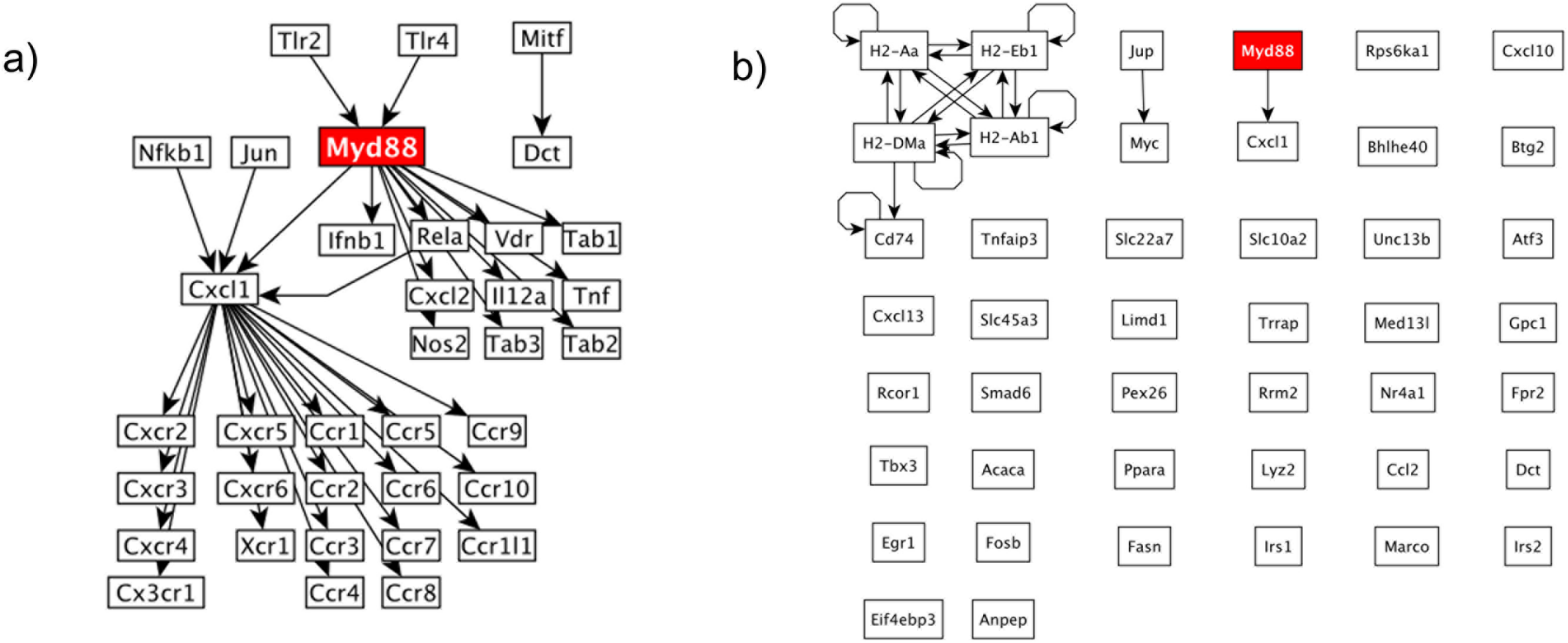
Figure shows the results for the dataset Myd88. The KO gene is shown in red in both panels. a) Mechanism results for HighEdgeS when we applied it on the Myd88 dataset. The results show two subgraphs. The one with the most genes shows the KO gene regulates the Cxcl1 gene and shows that the subgraph includes many Cxcl1 downstream genes. b) The results of the classical approach using DE genes. The Myd88 gene is connected to only one downstream gene. The results of the classical approach has many genes that are not connected indicating that the classical approach missed important interactions.

In this Myd88 knock-out dataset, the authors were interested in the effect of knocking out the Myd88 gene in liver [51], and the involvement of Myd88 in inflammatory response. The authors of the dataset reported that Myd88 is essential for Tnf survival mechanisms [51]. Further, they also reported the activation of Nfkb (aka Rela, shown in Fig 2a) by Myd88. From this dataset, HighEdgeS constructed a graph consisting of 35 edges and 36 genes where the KO gene plays a central role, as it can be seen in Fig 2a. In this result, HighEdgeS found many connected related genes from the same family, such as Cxcl, Tlr, and Tab. This graph shows the effect of Myd88 on Rela(Nfkb) and on Tnf, the same interactions reported in the experiment that produced the dataset.

The classical approach identified 20 edges and 44 genes. Although Myd88 was among them, the graph generated from the 42 genes, shown in Fig 2b, shows that most genes are not connected. Thus the classical results cannot really be used as a proposed putative mechanism. Among the genes identified by the classical approach, only the gene Cxcl1 was connected to the KO gene. Furthermore, the classical approach failed to identify the gene Rela, which was part of the known mechanism described by the authors of the experiment. Although the graph built using the results of the classical approach contains several interactions (shown in the top left corner of Fig 2b), none of the genes were connected to the known cause of phenomenon, the Myd88 gene.

**Fig 3:**
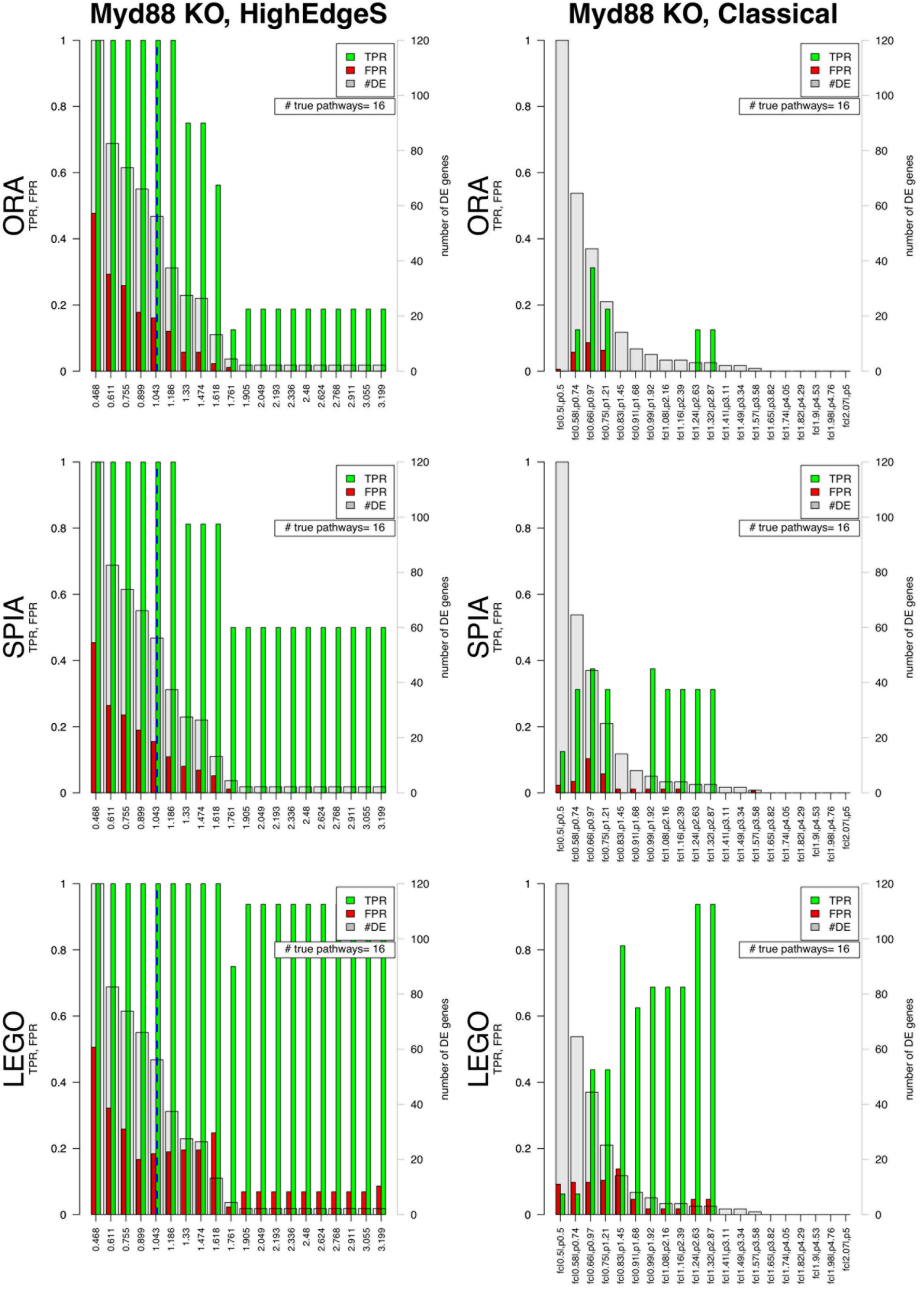
Comparison between the results from HighEdgeS (left columns) and the classical approach (right columns) for the Myd88 dataset. The three rows show the results of the pathway analysis methods ORA, SPIA, and LEGO. For each barplot, the x-axes show various values of the thresholds for each method. For the classical approach, the x-axes show various combinations of fold change and p-value thresholds. For the proposed approach the x-axes show the range from the point determined by change point analysis to the maximum value of edge scores. The y-axes in each graph show the scales for the false positive rate (FPR) and true positive rate (TPR). The right y-axes in each graph represent the number of DE genes shown by the gray bars. Blue dashed line represents the change point safety margin from edge point analysis (the default for the proposed method). In each bar plot, the green bars represent the TPR and red bars represent the FPR for pathways ranking when the significance threshold *α* = 0.1. True positive pathways are those containing the KO gene. The proposed method yields a perfect true positive rate of 100% in every case for its default threshold (blue dashed line). In contrast, the classical approach yields a TPR of less than 40% for all threshold combinations used with ORA (top right panel) and SPIA (middle right panel). The classical approach used with LEGO (lower right panel) yields a TPR varying between 0 and 93%. The figure also shows how the results of the classical approach depend very much on the combination of thresholds used for fold change and p-values, while the results obtained with the proposed method are much more stable.

When we applied the methods ORA, SPIA and LEGO to the list of genes obtained using the best choice of parameters from the classical approach, we obtained a TPR value of 0.31 and an FPR of 0.08 from ORA, a TPR value of 0.31 and an FPR of 0.01 from SPIA, and a TPR value of 0.93 and an FPR of 0.04 from LEGO. In contrast, while HighEdgeS obtained a TPR value of 1 and a FPR value of 0.16 when ORA was applied, a TPR value of 1 and a FPR of 0.15 from SPIA, and a TPR value of 1 and a FPR of 0.17 from LEGO (see Fig 3). The TPR yielded by GAGE was 1, and the FPR was 0.26. The TPR of GAGE is similar to the results of HighEdgeS. This shows that applying the methods ORA, SPIA, and LEGO on the mechanism found by HighEdgeS retrieves every single pathway truly involved with the phenotype (i.e. containing the KO gene), while yielding a number of false positives which is comparable with the classical analysis. The TPR value of 1 for HighEdgeS from this dataset is similar to the TPR from GAGE. However, the FPR values for the proposed method from ORA, SPIA and LEGO are better than FPR value of GAGE.

### NeuroD1 knock-out

In this experiment, the authors extracted mRNA from the mouse pineal gland, and then confirmed the KO using qRT-PCR [52]. NeuroD1 mRNA is highly abundant in the adult rat pineal gland and has been found to be involved with the regulation of insulin [52].

**Fig 4:**
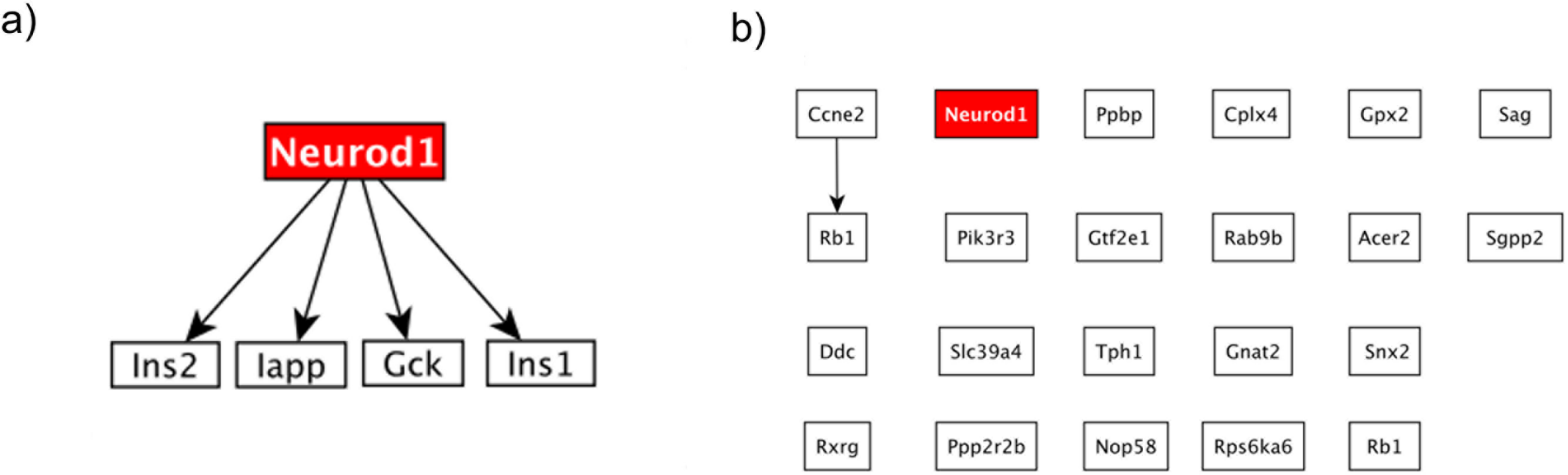
Figure shows the results for the dataset NeuroD1. The KO gene is shown in red in both panels. a) The mechanism found using HighEdgeS shows NeuroD1 is regulating Ins2, Iapp, Gck and Ins1. b) The results obtained using the classical approach for the same dataset. The classical approach results include the KO gene, but provides no explanation on how the suppression of this gene propagates further and affects the rest of the system.

When applied to this dataset, HighEdgeS constructs a network of 5 genes connected with 4 edges, shown in Fig 4a. In this network, the NeuroD1 gene is the root gene of the proposed putative mechanism, and has four genes immediately downstream of it: Ins1, Ins2, Iapp, and Gck. The presence of the Ins1 and Ins2 genes in this mechanism confirms the regulatory action of NeuroD1 on those genes. Studies demonstrated the effects of insulin on melatonin synthesis in the pineal gland through crosstalk between noradrenergic and insulin pathways [53]. This crosstalk also involves the gene Gck, involved in most glucose metabolism pathways [54].

When we applied the classical approach to the same dataset, we obtained a graph consisting of 22 genes and one edge, shown in Fig 4b. These results include a relatively small number of genes, yet when we inspect the graph constructed from these genes, we notice that the KO gene is not connected to any other gene. Thus, in this dataset as well, the classical approach is unable to yield any plausible mechanism for the given phenotype. Although the KO gene is shown in the result, this graph fails to propose any reasonable explanation on how the KO of NeuroD1 influenced the rest of the system.

When we applied ORA, SPIA, and LEGO to the networks obtained by the two approaches, the results of HighEdges yielded a TPR of 1 and a FPR of 0.08 from ORA, a TPR of 1 and FPR of 0.12 for SPIA, and TPR of 1 and FPR of zero for LEGO. The results of the classical approach yielded a TPR of zero and a FPR of zero for ORA, a TPR of 1 and FPR of 0 from SPIA, and TPR of 1 and FPR of 0 from LEGO. When we applied GAGE on the Neurod1 dataset, it yielded a TPR of 0, and a FPR of 0.04.

**Fig 5:**
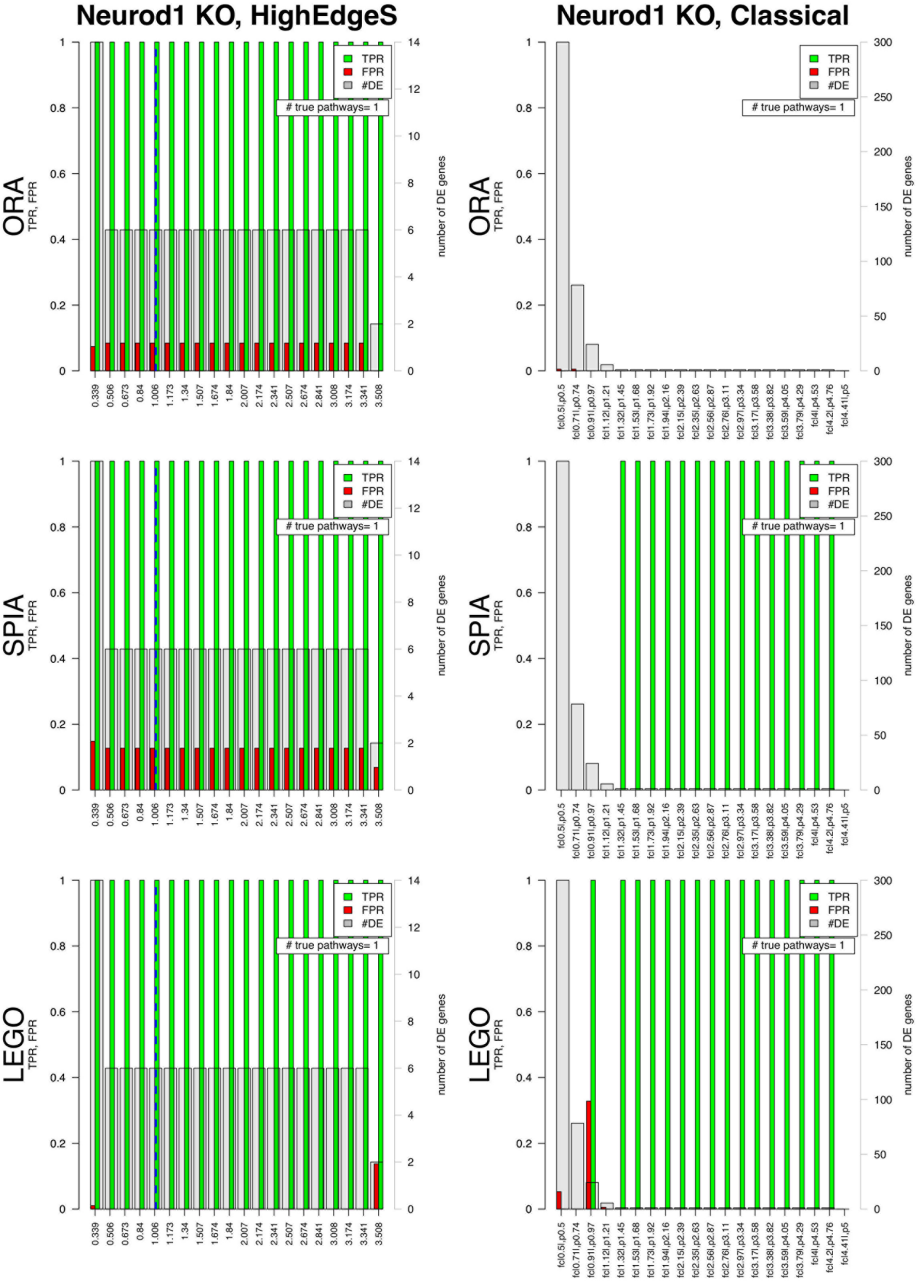
Comparison between the results from HighEdgeS (left columns) and the classical approach (right columns) for the Neurod1 dataset. The three rows show the results of the pathway analysis methods ORA, SPIA, and LEGO. For each barplot, the x-axes show various values of the thresholds for each method. For the classical approach, the x-axes show various combinations of fold change and p-value thresholds. For the proposed approach the x-axes show the range from the point determined by change point analysis to the maximum value of edge scores. The y-axes in each graph show the scales for the false positive rate (FPR) and true positive rate (TPR). The right y-axes in each graph represent the number of DE genes shown by the gray bars. Blue dashed line represents the change point safety margin from edge point analysis (the default for the proposed method). In each bar plot, the green bars represent the TPR and red bars represent the FPR for pathways ranking when the significance threshold *α* = 0.1. True Positive pathways are those containing the KO gene. The classical approach yields a TPR of 0 for all threshold combinations used with ORA (top right panel). For SPIA and LEGO, the classical approach yields 0 true positives for the more lenient thresholds, to the left of the x axis. For the more stringent thresholds, in this dataset there is only one DE gene left, the KO gene, which in turn identifies the true positive pathway. As explained in the text, the correct threshold is never known a priori which means that the classical approach may or may not identify the correct pathway, depending on the choice of the threshold. In contrast, the proposed method yields a perfect true positive rate of 100% in every case for its default threshold that is calculated automatically (blue dashed line).

This means that no pathway involved with the phenotype was reported as significant when analyzing with the list of genes obtained from the classical approach, when ORA was applied yet with SPIA and LEGO the TPR was one. At the same time, the analysis of SPIA, LEGO, and ORA of the results yielded by HighEdgeS is able to identify as significant the only pathway truly involved with the phenotype (see Fig 5). The TPR value of 1 for HighEdgeS in combination with any of the ORA, SPIA and LEGO is substantially better than the TPR from GAGE. In essence, the proposed method is able to correctly find the only pathway containing the knockout gene regardless of the method used to identify the significant pathways. At the same time, GAGE fails to identify the causal pathway in this data set.

### Pdx1 knock-out

**Fig 6:**
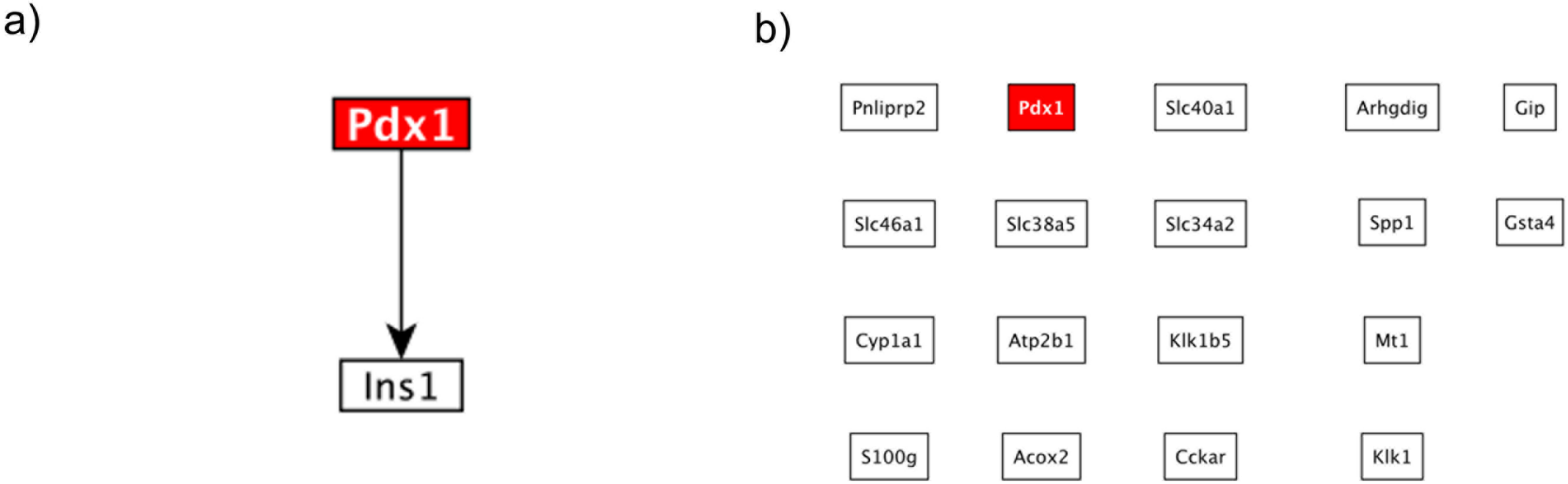
The comparison between the putative mechanisms constructed by HighEdgeS and the classical approach for the Pdx1 dataset. a.) The putative mechanism constructed by HighEdgeS. The graph shows the knocked out gene Pdx1 regulating the Ins1 gene. b.) The results for the same dataset when the classical approach was applied. The results of the classical approach lack any connection among the genes and the KO. However, the results of the proposed approach show the interaction between the Pdx1 gene and the Ins1. In fact, the authors of the dataset discussed this interaction in their work indicating the role of the Ins1 in this condition.

In this dataset the authors were interested in the effect of the knock-out of the transcription factor Pdx1 and its role in the duodenum [55]. When we applied HighEdgeS to analyzed this dataset, it produced a connected graph with only two genes, the KO gene and the gene Ins1. When applied to this dataset, the classical approach reported 18 DE genes, including the KO gene. However, no known interaction was found among these genes, as shown in Fig 6b, showing that the classical approach failed again to propose a meaningful mechanism able to explain the given phenotype.

**Fig 7:**
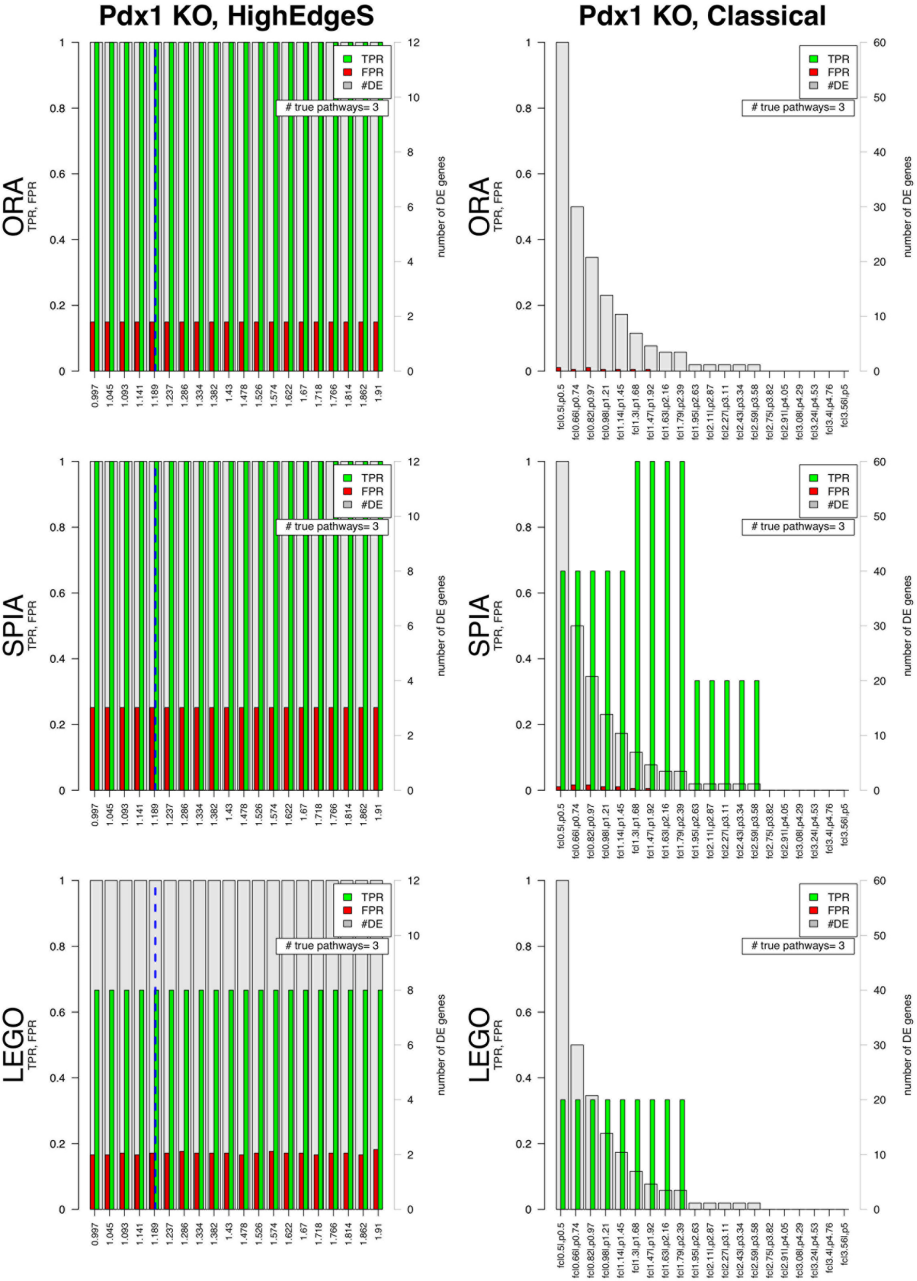
Comparison between the results from HighEdgeS (left columns) and the classical approach (right columns) for the Pdx1 dataset. The three rows show the results of the pathway analysis methods ORA, SPIA, and LEGO. For each barplot, the x-axes show various values of the thresholds for each method. For the classical approach, the x-axes show various combinations of fold change and p-value thresholds. For the proposed approach the x-axes show the range from the point determined by change point analysis to the maximum value of edge scores. The y-axes in each graph show the scales for the false positive rate (FPR) and true positive rate (TPR). The right y-axes in each graph represent the number of DE genes shown by the gray bars. Blue dashed line represents the change point safety margin from edge point analysis (the default for the proposed method). In each bar plot, the green bars represent the TPR and red bars represent the FPR for pathways ranking when the significance threshold *α* = 0.1. True Positive pathways are those containing the KO gene. The figure shows that the proposed approach yields a higher TPR than the classical approaches overall. For ORA, the classical approach fails to identity any of the 3 pathways containing the KO gene independently of the threshold combination used (top right panel). The classical approach used with SPIA identifies 1, 2 or 3 of the 3 true positive pathways depending on the threshold combination used (middle right panel). Finally, the classical approach used with LEGO identifies only one of the 3 true positive pathways and that only for about 1/3 of the range of thresholds explored. In contrast, the proposed approach used with ORA (top left) and SPIA (middle left) identifies all true positive pathways for any edge scores. When combined with LEGO (bottom left), the proposed approach identifies 2 out of the 3 true positives, still much better than the classical approach with LEGO (bottom right).

When ORA was applied on the list of genes produced by HighEdgeS, yielded a TPR of 1 and an FPR of 0.14, a TPR of 1 and FPR of 0.25 from SPIA, and a TPR of 0.66 and FPR of 0.17 from LEGO. The list of genes produced by the classical approach yielded a TPR of zero and an FPR of 0.01 for ORA, a TPR of 1 and FPR of zero from SPIA, and a TPR of zero and FPR of zero from LEGO (see Fig 7). The TPR yielded by GAGE was 0.66, and the FPR was 0.15.

In this case, although the classical approach correctly found the KO gene among the DE genes, the presence of other genes, not relevant to the phenomenon, negatively impacted the results of ORA, *de facto* hiding the real phenomenon. Interestingly, HighEdgeS produced the same result independently of the value used for the safety margin, indicating that the method is very stable with respect to the choice of the parameters. The results of SPIA on the list of genes from the classical approach were perfect (no false positive) and identified all true pathways. When combined with ORA and SPIA, HighEdgeS yielded a perfect TRP of 1, identifying all 3 pathways containing the knockout gene. When combined with LEGO, the proposed method identified 2 of the 3 causal pathways, GAGE only identified 2 of the 3 causal pathways, as well. The processed data and the code used to obtain the results in this section can be found in (S1 Folder).

## Discussion

Identifying the mechanisms involved in a disease is an important step toward understanding diseases and developing effective treatments. Many approaches that aim to identify these mechanisms use a selection based on thresholds on differential expression or p-value to find genes of interest in the given phenotype. These approaches ignore the interactions between genes, and they are very sensitive to threshold selection [56, 57]. Here we described a method that overcomes these limitations, and produces results that are more biologically relevant and less sensitive to the choice of parameters, when compared to the results of the classical approach.

The classical approach uses two different measures, fold change and p-values. The fold changes are often considered on a log scale and the p-values are usually corrected for multiple comparisons with methods such as FDR. However, the user is still required to make two choices: for the log fold change and for the FDR-corrected p-value. As Figs 3, 5, and 7 show, the results can vary dramatically depending on the choices made for these parameters. The proposed method has a single parameter, the edge scores, and the approach calculates the best value for this threshold in each case. Thus, the user does not need to make any choices whatsoever. The results in Figs 3, 5, and 7 (blue line in each left panel) show this calculated threshold provides better results than most of the choices based on fold change and p-values in the classical approach. Since the goal of our method is to identify the mechanism in a phenotype comparison, the only way in which such a method can be truly validated is to analyze data from experiments satisfying two conditions: i) the cause of the phenotype is known, and ii) the cause of the phenotype is related to a single gene, rather than a combination of genes, or gene-environment interaction(s). This is why we only looked for knockout (KO) datasets. Furthermore, the proposed method is using gene-gene interactions from the signaling pathways of the KEGG database. Thus, to evaluate our method with any dataset, the dataset must come from a KO experiment and the knocked-out gene must be connected with other gene(s) in the signaling pathways of KEGG. At this time, mouse is the only organism that has data from single cause experiments, that are of sufficient quality, and that involve sufficiently well annotated genes to allow us to prove the mechanisms found are correct (see S1 File for more details). Even though the validity of the method could only be demonstrated on mouse data, there is nothing specific to mouse in the analytical approach used and the method can be applied to any organism, as well as it is reasonably well annotated.

In all three datasets illustrated here, the proposed method was able to identify interactions that both: i) were known to be related to the phenomenon, and ii) constitute a plausible explanation for how the effect of the KO gene was propagated in the rest of the system. At the same time, the classical approach mostly produced disconnected graphs that were not helpful to explain other changes in the organism.

A quick look at the results of HighEdgeS might suggest that it was simply reporting all the genes connected with a gene of a large fold change (FC). This is not the case. In the Myd88 dataset, the Myd88 gene is directly connected to 35 downstream genes and 13 upstream genes in the KEGG global graph (the global graph is described in section Materials and Methods). In the results of the Myd88 dataset HighEdgeS reported only 10 downstream genes and only 2 upstream genes as shown in Fig 2a. This is a concrete example demonstrating that the proposed method does not report genes just because they are connected with a gene of a large FC.

Table 1 summarizes the comparison between the results of HighEdgeS, the classical approach, and GAGE as they would be used in practice. Three pathway analysis methods (ORA, SPIA and LEGO) were applied on the genes yielded by HighEdgeS and the classical approach. GAGE uses the entire set of genes to directly identify pathways so ORA, SPIA and LEGO are not necessary for GAGE. For the classical approach we selected the DE genes using an absolute fold change greater than 2 and FDR-corrected p-value less than 0.05. For the Myd88 and Neurod1 datasets, no genes meet these thresholds showing the limitations of this approach. The performance was measured using the true positive rate (TPR) and false positive rate (FPR) for each data set. The results show that HighEdgeS yields the best TPR for all datasets. HighEdgeS also yields the best FPR for 2 out of the 3 datasets. It is important to note that a perfect TPR (or sensitivity) can always be obtained if the results include all pathways. This, of course, will come with a very poor FPR since all but the true positives pathways will be false positives. Conversely, a perfect FPR (or specificity) can always be obtained if the results do not include any pathway. This however, will be associated with a TPR of zero since no true positives will be included in the results. This is precisely what happens for the classical approach for the Pdx1 dataset. The classical approach has a lower FPR values, only because its sensitivity is very low (not being able to identify any true positive (TP) pathway in 2 out of 3 cases, and identifying only one of the 3 true positive pathway in the remaining case). In contrast, HighEdgeS identified all true positive pathways in 2 out of 3 cases (when combined with ORA and SPIA) and 2 out of the 3 TP pathways when combined with LEGO.

**Table 1:** A comparison between the results of HighEdgeS, the classical approach, and GAGE as they would be used in practice. Three pathway analysis methods (ORA, SPIA and LEGO) were applied on the genes yielded by HighEdgeS and the classical approach. GAGE uses the entire set of genes to directly identify pathways so ORA, SPIA and LEGO are not applicable for GAGE. The green background indicates the best results obtained for each dataset (each row). For the classical approach we selected the DE genes using an absolute fold change greater than 2 and FDR-corrected p-value less than 0.05. “No DE” means no genes met those thresholds. The performance was measured using the true positive rate (TPR) and false positive rate (FPR) for each data set. The results show that HighEdgeS yields the best TPR for all datasets. HighEdgeS also yield the best FPR for 2 out of the 3 datasets. Furthermore, for the Pdx1 dataset, even though the classical approach has a lower FPR values, its sensitivity is very low (not being able to identify any true positive (TP) pathway in 2 out of 3 cases, and identifying only one of the 3 true positive pathway in the remaining case). In contrast, HighEdgeS identified all true positive pathways in 2 out of 3 cases (when combined with ORA and SPIA) and 2 out of the 3 TP pathways when combined with LEGO.

		HighEdgeS			Classical			GAGE
		ORA	SPIA	LEGO	ORA	SPIA	LEGO	
TPR	Myd88	1	1	1	No DE	No DE	No DE	1
	Neurod1	1	1	1	No DE	No DE	No DE	0
	Pdx1	1	1	0.66	0	0.33	0	0.66
FPR	Myd88	0.16	0.15	0.17	No DE	No DE	No DE	0.26
	Neurod1	0.08	0.12	0	No DE	No DE	No DE	0.04
	Pdx1	0.14	0.25	0.17	0	0	0	0.15

It is important to note that some of the existing pathway annotations may have some annotation bias. For instance, the biochemical pathways in KEGG are acknowledged to have some bias toward metabolism [58]. Other recent work also shows that few pathways that contain very well studied genes tend to appear as significant in many enrichment analyses just because they are involved in very well studied diseases such as cancer [41]. Also, the degree to which a given genome is annotated in various databases including KEGG is another important potential limitation of this approach. More specifically, since this approach relies on existing annotations, the proportion of well studied genes in a given genome is important. Better results are more likely to be obtained for data from human or other common model organisms such as mouse, rat, drosophila, etc. Since the interactions that we are using to get the results of our method are limited to the genes in KEGG, this might be a limitation to this approach. Nevertheless, annotation bias will affect any type of analysis approach. We expect that the results provided by the method proposed here will become more and more accurate as the sources of various annotations—including KEGG—become more accurate themselves and start to reduce some of their existing intrinsic bias.

## Supporting information

S1 File Search for knockout datasets. (PDF)

S1 Folder Code and data. Folder with the code and dataset used in the paper.
(ZIP)
