# Supplementary material for: Identifying biologically relevant putative mechanisms in a given phenotype comparison
Source: PLoS One. 2017 May 9;12(5):e0176950. doi: 10.1371/journal.pone.0176950 (PMC5423614; doi:10.1371/journal.pone.0176950)
Supplement: S1 File — (PDF) [file pone.0176950.s001.pdf]

Supplementary Materials

January 11, 2017

Search for knockout datasets

We looked for data sets in both NCBI's GEO [1] and EBI's ArrayExpress [2]. The NCBI GEO contains 309 knockout datasets.[1] Out of the 309 knockout datasets, 290 were mouse, 10 were human, and 9 were other organisms. For the 10 human datasets, 9 datasets did not have the KO gene in KEGG. The last dataset, GSE2781, involves both human and mouse cells and the KO was actually performed in the mouse samples. For the yeast (*Saccharomyces cerevisiae*), the dataset GSE8761 has several KO genes in the Ribosome pathway, yet none of them were connected with any other gene. The same is with the dataset GSE2683. The KO gene in the GSE1938 dataset is present in some KEGG signaling pathway but not connected to any other genes. For *Arabidopsis thaliana*, the KO gene was not in any KEGG signaling pathway for all three datasetes. For *Plasmodium falciparum*, the two KO genes EBA140 and EBA175 of the dataset GSE3877 were knocked out and found in the malaria KEGG pathway (pfa05144). When we checked the file (pfa05144.xml) which contains the genes interactions information in KEGG pathways the two genes were not connected with any other genes in the Malaria pathway. As a result, we could not use this dataset. The other dataset GSE2369 for the species *Plasmodium falciparum* the KO gene was not found in any KEGG signaling pathway. For the *Pan troglodytes* species dataset GSE62572, there was no KO gene. The researchers used as special medium called "knockout serum replacement". For *Macaca mulatta* dataset GSE8749, *Pseudomonas aeruginosa* dataset GSE2885, and *Escherichia coli* K-12 dataset GSE9814, the respective KO genes were not in any KEGG signaling pathway.

We also search the EBI ArrayExpress website.[2] This search returned 606 knockout experiments of which 471 of them were performed on mouse only, 26 on human (tissues or cell lines) only, 8 on mouse and human, 33 on arabidopsis, 1 on mouse and *Mus spretus* , 1 on mouse, human and *Rattus norvegicus*, 1 on *Candida albicans* and *Candida parapsilosis* and 65 on other organisms (each dataset for one organism). Out of the 35 (26+8+1) datasets involving KO experiments on human tissues, in 17 cases the KO gene was not found in any KEGG signaling pathway. In 5 such datasets the experiment involved multiple species (e.g. mouse, human), and in all 5 datasets the KO was performed on the mouse samples. In three datasets the KO gene was miRNA not RNA. In another 3 datasets, the KO genes were found in KEGG but not connected with an interaction edge in the signaling pathway. In 2 datasets the gene of interest was downregulated but not knocked out. One dataset has KO samples with no control samples to compare with, and in another dataset the KO gene was connected in KEGG to miRNA not RNA. There were three datasets that the KO gene was found in KEGG and connected

[1] Obtained by searching the DataSet Browser `https://www.ncbi.nlm.nih.gov/sites/GDSbrowser/` with the term *"knockout"*

[2] `https://www.ebi.ac.uk/arrayexpress/` searched with *evv:"knockout" and raw:true and microarray, Filtered by experiment type "rna assay", experiment type "array assay".*

to at least one edge. In the dataset E-GEOD-84693 the gene STAT1 was supposed to be knocked out. When we processed this dataset, the fold change of the STAT1 gene showed that STAT1 was far from being knocked out (it had an actual small increase of 0.2 fold change). The measured values showed that the experiment actually failed to knockout STAT1 and therefore, it would be futile to look for mechanisms. In the dataset E-GEOD-54830, the target of the KO experiment, BRCA2, was in the KEGG signaling pathways and connected. However, BRCA2 was ranked 36th based on p-values. This shows that the experiment greatly disturbed a large number of other genes, thus not satisfying our requirement of having a single point cause. The third dataset that we analyzed was E-GEOD-35973 that has the KRAS gene knocked out. In this dataset the researchers compared samples for cells with K-ras mutant versus KRAS knockout, without a control. When we analyzed this dataset the fold change for the KRAS gene was -0.13 which is expected since the gene is perturbed/disrupted in both groups. While this experiment confirms that the given mutation KRAS shuts down the activity of KRAS as much as a knockout, it would not allow us to search for a mechanism since there is no control to compare with.

For the *Arabidopsis thaliana*, there were 33 datasets available in EBI. In 28 out of the 33 datasets the KO gene was not in KEGG. For the other 5 the .db file was not available at the BRAINARRAY database which we used for the custom chip definition files (CDF). We used these custom CDFs for the preprocessing of the microarray data because they have been shown to improve the precision and accuracy of gene expression measurements [3]. For the *Candida albicans* species 7 datasets were found. In 6 out of the 7 datasets the KO gene was not in KEGG, and the 7th dataset the *Candida albicans* the microarray chip was not supported by the BRANINARRAY database. For the *Candida parapsilosis* the KO was on the *Candida parapsilosis* species. For the *Danio rerio* species 1 dataset was found, the KO gene was miRNA not RNA which our method currently does not support dataset with miRNA genes. Two datasets were found for the *Escherichia coli* species, two of them the strains were not supported by KEGG. Thus we could not look for the KO in KEGG. For the *Halobacterium sp. NRC-1* species 3 datasets were found, 2 out of the 3 the KO genes were found in KEGG metabolism pathway not in a signaling pathway. The other dataset the KO gene was not in any KEGG pathways. One dataset was found for each of the following species: *Acinetobacter baumannii*, *Anopheles gambiae*, *Bacillus anthracis*, *Caenorhabditis elegans*, *Mycobacterium tuberculosis* ,*Mycobacterium smegmatis*, *Helicobacter pylori* P12, *Pimephales promelas*, *Oryza sativa*, and *Oryza sativa Japonica Group.* Two datasets were found for each of the following species: *Plasmodium berghei*, *Physcomitrella patens*, *Mycobacterium tuberculosis* CDC1551, *Gallus gallus.* Six datasets involving *Drosophila melanogaster* were found. In each of these datasets from these species the KO gene was not found in any of the KEGG signaling pathways.

One dataset for the following species were found in the EBI ArrayExpress database: *Plasmodium falciparum* 3D7, *Schizosaccharomyces pombe*, *Staphylococcus aureus subsp*, *Pseudomonas aeruginosa*, *Pseudomonas putida* KT2440, *Kluyveromyces lactis*, *Streptococcus mutans* UA159, *Streptococcus pyogenes* and *Toxoplasma gondii.* Two datasets for the following species were found: *Salmonella enterica subsp*, *Sus scrofa*, and *Neisseria meningitidis*, were found. All these datasets the microarray platform used was not supported with the BRAINARRAY database. Three datasets for *Rattus norvegicus* species were found. The first one E-GEOD-44962 it has 3 KO genes, for the two gene BCRP and MRP2 the genes are not connected to other genes and the Mdr1a gene was not in any KEGG signaling pathway. The second dataset E-GEOD-77377 uses a rat microarray that is not supported in the BRAINARRAY database. The third dataset E-GEOD-8316 the KO was performed on mouse samples (Note: this dataset has human and mouse samples). One dataset was found for the *Mus spretus* the KO was performed on mouse samples. For the *Saccharomyces cerevisiae* 10 datasets were found, two of them the 2 KO genes were not in KEGG signaling pathways, the other 4 microarray chips were not supported in the BRAINARRAY database, and the other 4 datasets the BRAINARRAY database was missing the .db file required for processing the data.

In summary, none of the 19 non-mouse datasets from the NCBI GEO database, or the 123 non-mouse datasets from the EBI ArrayExpress can be used to prove that whatever mechanism we would find would be the correct one. In spite of our extensive search, at this time, mouse is the only organism that has data from single cause experiments, that are of sufficient quality, and that involve sufficiently well annotated genes to allow us to prove the mechanisms found are correct.

References

[1] Barrett T, Suzek TO, Troup DB, Wilhite SE, Ngau WC, Ledoux P, et al. NCBI GEO: mining millions of expression profiles–database and tools. Nucleic Acids Research. 2005;33(Database Issue):D562–6.

[2] Parkinson H, Sarkans U, Shojatalab M, Abeygunawardena N, Contrino S, Coulson R, et al. ArrayExpress–a public repository for microarray gene expression data at the EBI. Nucleic Acids Research. 2005;33(Database issue):D553–D555.

[3] Sandberg R, Larsson O. Improved precision and accuracy for microarrays using updated probe set definitions. BMC bioinformatics. 2007;8(1):1.
